# Neural Correlates of Early Sound Encoding and their Relationship to Speech-in-Noise Perception

**DOI:** 10.3389/fnins.2017.00479

**Published:** 2017-08-25

**Authors:** Emily B. J. Coffey, Alexander M. P. Chepesiuk, Sibylle C. Herholz, Sylvain Baillet, Robert J. Zatorre

**Affiliations:** ^1^Department of Neurology and Neurosurgery, Montreal Neurological Institute, McGill University Montréal, QC, Canada; ^2^Laboratory for Brain, Music and Sound Research Montréal, QC, Canada; ^3^Centre for Research on Brain, Language and Music Montréal, QC, Canada; ^4^German Center for Neurodegenerative Diseases Bonn, Germany; ^5^McConnell Brain Imaging Centre, Montreal Neurological Institute, McGill University Montréal, QC, Canada

**Keywords:** frequency-following response, speech-in-noise, magnetoencephalography, electroencephalography, neuroplasticity, auditory perception, inter-individual variability, musical training

## Abstract

Speech-in-noise (SIN) perception is a complex cognitive skill that affects social, vocational, and educational activities. Poor SIN ability particularly affects young and elderly populations, yet varies considerably even among healthy young adults with normal hearing. Although SIN skills are known to be influenced by top-down processes that can selectively enhance lower-level sound representations, the complementary role of feed-forward mechanisms and their relationship to musical training is poorly understood. Using a paradigm that minimizes the main top-down factors that have been implicated in SIN performance such as working memory, we aimed to better understand how robust encoding of periodicity in the auditory system (as measured by the frequency-following response) contributes to SIN perception. Using magnetoencephalograpy, we found that the strength of encoding at the fundamental frequency in the brainstem, thalamus, and cortex is correlated with SIN accuracy. The amplitude of the slower cortical P2 wave was previously also shown to be related to SIN accuracy and FFR strength; we use MEG source localization to show that the P2 wave originates in a temporal region anterior to that of the cortical FFR. We also confirm that the observed enhancements were related to the extent and timing of musicianship. These results are consistent with the hypothesis that basic feed-forward sound encoding affects SIN perception by providing better information to later processing stages, and that modifying this process may be one mechanism through which musical training might enhance the auditory networks that subserve both musical and language functions.

## Introduction

Understanding the neural bases of good speech-in-noise (SIN) perception during development, adulthood, and into old age is both clinically and scientifically important. However, it is challenging due to the complexity of the skill, which can be considered as a special case of auditory scene analysis, and can involve multiple cognitive processes depending on the information that is offered including spatial location, spectral and temporal regularity, and modulation (Moore and Gockel, 2002; Pressnitzer et al., 2011), and can be aided by visual cues (Suied et al., 2009), by predictions formed with the motor system (Du et al., 2014), and based on prior knowledge such as of language (Pickering and Garrod, 2007; Golestani et al., 2009) that can be used to constrain the interpretation of noisy information (Bendixen, 2014). The contribution of the fidelity with which an individual encodes various sound properties, including periodicity, which varies according to a variety of life experiences (e.g., musical experience Anderson et al., 2013a) is not yet clear.

One means of observing the inter-individual differences in how people encode periodic characteristics of sound is the frequency-following response (FFR), an evoked response that is an index of the temporal representation of periodic sound in the brainstem (Chandrasekaran and Kraus, 2010; Skoe and Kraus, 2010), thalamus, and auditory cortex (Coffey et al., 2016b,c). Differences in the strength and fidelity of the fundamental frequency (f0) of the FFR have been linked to SIN perception such that increased FFR amplitude is associated with better performance (reviewed in Du et al., 2011, see also Parbery-Clark et al., 2009a; Anderson et al., 2012). However, enhancements and deficits of neural correlates that are related to SIN perception are most consistently observed either when the FFR is measured in very challenging listening conditions (e.g., Parbery-Clark et al., 2009b), in the degree of degradation of the FFR signal between quiet and noisy conditions (e.g., Cunningham et al., 2001; Parbery-Clark et al., 2011a; Song et al., 2011), or in the magnitude of enhancement that is conferred by predictability within a sound stream (e.g., Parbery-Clark et al., 2011c). f0 representation in the FFR may be enhanced by training (Song et al., 2008, 2012) and is often observed to be stronger among musicians even to sounds presented in silence (e.g., Musacchia et al., 2007), suggesting that learning mechanisms related to identifying task-relevant features and possibly attention might act to bias and enhance incoming acoustic information and suppress noise (Suga, 2012).

However, a clear picture has not yet emerged (Coffey et al., 2017). It is unclear if the sometimes-observed relationship between the FFR and SIN is due *only* to better top-down mechanisms such as better stream segregation (Başkent and Gaudrain, 2016) or selective auditory attention (Parbery-Clark et al., 2011b; Song et al., 2011; Lehmann and Schönwiesner, 2014), or if enhanced feed-forward stimulus encoding also plays a role. Here, we aimed to better understand the neural bases of periodicity coding in the brain under optimal listening conditions to understand its relevance to SIN; if basic encoding of sound quality in silence is important for more complex tasks such as hearing in noise, then we predict that there should be a relationship between FFR measured in silence and SIN performance. A secondary question we address is whether this relationship might be enhanced by musicianship.

Musicians are thought to have both enhanced bottom-up (Musacchia et al., 2007; Bidelman and Weiss, 2014) and top-down (Strait et al., 2010; Kraus et al., 2012) processing of sound. Because SIN perception and measures of basic sound encoding are related to musicianship, musical training has been proposed as a means of ameliorating poor SIN performance (reviewed in: Alain et al., 2014). Musical training places high demands on sensory, motor, and cognitive processing mechanisms that overlap between music and speech perception, and offers extensive repetition and emotional reward, which could stimulate auditory system enhancements that in turn impact speech processing (Patel, 2014). Several longitudinal studies support a causal relationship between musical training and SIN skills (Tierney et al., 2013; Kraus et al., 2014; Slater et al., 2015), although it is difficult to maintain full, experimental control over naturalistic training studies (Evans et al., 2014). A number of cross-sectional studies have also reported a musician advantage in SIN perception (Parbery-Clark et al., 2009a,b, 2011b, 2012b; Strait et al., 2012; Zendel and Alain, 2012; Swaminathan et al., 2015); however, other studies have not found significant group differences (Ruggles et al., 2014; Boebinger et al., 2015) or have found the musicianship effect to be dependent upon the specific SIN task variations, such as the degree of information masking (Swaminathan et al., 2015; Başkent and Gaudrain, 2016) or the degree of reliance on pitch cues (Fuller et al., 2014). A recent review of SIN perception among musicians concluded that on balance there is good evidence for musician enhancement of SIN, but also highlighted the diversity of study designs used to study hearing in noisy conditions, which may contribute to inconsistent findings (Coffey et al., 2017). However, it remains uncertain to which aspects of cognition any musician advantage is owed: top-down processes such as selective attention and working memory that modulate early levels (Rinne et al., 2008) to filter and temporarily store incoming information (Strait and Kraus, 2011; Kraus et al., 2012), relatively immutable factors such as non-verbal IQ (Boebinger et al., 2015) that might affect multiple cognitive processes, or differences in basic sound encoding (reviewed in Anderson and Kraus, 2010b; Du et al., 2011; Alain et al., 2014, see also Weiss and Bidelman, 2015).

In the present study, we first aimed to clarify whether robust f0 encoding in the auditory system, which is known to be enhanced in musicians (Musacchia et al., 2007; Bidelman et al., 2011a,b), influences SIN perception in a feed-forward fashion. Rather than relating fundamental encoding recorded in the presence of noise to later performance (which has previously been shown, described above) and might include influences from top-down processes that spontaneously act to separate speech and noise streams, here we reduce the similarity between the conditions of the electrophysiological recording and the offline SIN behavioral task to a single overlapping feature: the presence of pitch-related information. Although several studies have not found a significant relationship between FFR-f0 measured in conditions of silence and SIN performance (e.g., Parbery-Clark et al., 2009a), such relationships may be obscured by EEG-based FFR recordings which likely blend responses coming from different sources (Zhang and Gong, 2016; Tichko and Skoe, 2017). Despite the relative insensitivity of magnetoencephalography (MEG) to deep sources (which approximate radial sources, Baillet et al., 2001), sufficient information is preserved in the MEG signal for accurate localization of deeper structures such as the hippocampus, amygdala and thalamus (Attal and Schwartz, 2013; Dumas et al., 2013), and for the contributions from subcortical and cortical FFR generator sites to be separated (Coffey et al., 2016b), which may increase the sensitivity of the experimental design to behavioral relationships. Therefore, an additional novel aspect of the present study is to use MEG to determine how FFR signals coming from distinct anatomical structures may be contributing to the putative relationship to SIN performance. We thus extend previous investigations of the anatomical origins of FFR signals to their behavioral meaning in the context of SIN perception.

If enhanced encoding is partly responsible for better auditory skills because a better quality signal is encoded from incoming sound and passed to higher-order cognitive processes and networks (Irvine, 1986; Musacchia et al., 2008), we would expect that the relationship between SIN and sound encoding would persist even under optimal listening conditions when the system is not challenged, and when the listener's attention is otherwise engaged. We therefore first measured SIN perception behaviorally, then in a separate session simultaneously recorded EEG and MEG data while listeners were presented with a speech sound in quiet as they watched a silent film. Secondarily, we evaluate correlations of measures of musical experience with FFR-f0 and SIN within our sample to evaluate the possible influence of musicianship on performance.

In addition to FFR-f0, which is derived from the higher-frequency EEG activity (Skoe and Kraus, 2010), other lower-frequency cortical potential measures covary with SIN performance, in particular the ERP P2 component (~200 ms post stimulus onset; Cunningham et al., 2001), which is also known to be related to speech processing and is sensitive to training effects (Key et al., 2005; Musacchia et al., 2008; Bidelman and Weiss, 2014; Tremblay et al., 2014). If the two signals represent sequential processes in the same processing stream, we would expect enhancements in FFR-f0 to be paralleled by enhancements in the strength of the ERP P2 component, and for each of these measures to be related to SIN accuracy. To test this hypothesis, we used distributed source modeling of the magnetic signals to localize the neural origins of the MEG FFR-f0 and the P2, and examined their spatial and statistical relationships to each other (as well as spatial relationships to preceding and following ERP components) for the first time in order to explore how these signals may be related. Collectively, these data should help us to understand the neural basis of inter-individual differences in sound encoding and its effects on the important real-world function of SIN perception.

## Methods and materials

The experimental procedures concerning the MEG and (single channel, Cz) EEG recordings of the brain's response to the speech syllable /da/, and much of the pre-processing, have previously been reported in the context of determining their neural origins and will be discussed only briefly here (please see Coffey et al., 2016b “Methods” for details). The correlations between FFR-f0 strength and musicianship that are included in the summary of musical enhancements in Table 1 have been reported in Coffey et al.; all other findings have not been reported previously. Behavioral testing took place in a sound-attenuated room on different day prior to the MEG recording session.

**Table 1:** Summary of evidence for musicianship-related behavioral and neurophysiological enhancements (*N* = 12).

**Measure**	**Age of start**	**Practice hours**
SIN	rs = −0.70, *p* = 0.006^*^	rs = 0.39, *p* = 0.10
Fine pitch discrimination	rs = 0.45, *p* = 0.07	rs = −0.67, *p* = 0.008^*^
FFR-f0 (right AC)	rs = −0.53, *p* = 0.05^*^	rs = 0.57, *p* = 0.04^*^
P2 amplitude	rs = −0.21, *p* = 0.25	rs = 0.59, *p* = 0.05^*^

Asterisks (^*^) indicate significant rank correlations (alpha < 0.05, one tailed). In general, earlier start ages and a larger number of practice hours are associated with enhancements, suggesting an influence of musical training. Note that lower fine pitch discrimination scores indicate better performance; therefore correlations in opposite directions are expected.

### Participants

Data from the same 20 neurologically healthy young adults included in the previous study (Coffey et al., 2016b) were included in this study (mean age: 25.7 years; *SD* = 4.2; 12 female; all were right-handed and had normal or corrected-to-normal vision; < = 25 dB hearing level thresholds for frequencies between 500 and 4,000 Hz assessed by pure-tone audiometry; and no history of neurological disorders). All but three subjects were native English speakers; the other three (one Korean, two French speakers) were highly proficient in English and all scored within the range of the native speakers on the HINT task, thus ruling out that any of our findings were due to second-language effects. Informed consent was obtained and all experimental procedures were approved by the Montreal Neurological Institute Research Ethics Board.

### Speech-in-noise assessment

SIN was measured using a custom computerized implementation of the hearing in noise test (HINT; Nilsson, 1994) that allowed us to obtain a relative measure of SIN ability using a portable computer, without specialized equipment. In the standard HINT task, speech-spectrum noise is presented at a fixed level and sentences are varied in a staircase procedure to obtain a (single-value) SIN perceptual threshold (Nilsson, 1994). Our modified HINT task used a subset of the same sentence lists (Bench et al., 1979) and speech-spectrum noise, but presented thirty sentences in three empirically determined difficulty levels in randomized order: easy (2 dB SNR; i.e., target speech was 2 dB louder than noise), medium (−2 dB SNR), and difficult (−6 dB SNR). The sentences and noise were combined using sound processing software (Audacity, version 1.3.14-beta, http://audacity.sourceforge.net/; 44100Hz sampling frequency). Stimuli were presented diotically (i.e., identical speech and masker in each ear) via headphones (JVC HA-M5X) with the noise adjusted to a loud but not uncomfortable sound level on pilot subjects (~75 db SPL) and thereafter held constant. No verbal or visual feedback was given. A single overall accuracy score as the proportion of sentences correctly repeated back to experimenter was calculated by averaging the accuracy across all three levels; however, the score distributions showed a clear ceiling effect in the easiest level, with 12 out of 20 participants scoring over 95% (mean of subset of easy trials: 93.5%, *SD* = 7.5; medium trials: 79.9%, *SD* = 14.0; hard trials: 32.6%, *SD* = 12.1). We therefore excluded the easiest trials from the mean accuracy score in order to obtain a cleaner estimate of inter-individual variability; control analyses were conducted for the main research questions by assessing correlations between SIN scores at each level of difficulty and the FFR-f0 strength to ensure that the pattern of results was robust to this exclusion.

### Fine pitch discrimination

Fine pitch discrimination thresholds were measured as described in Coffey et al. (2016b), using a two-interval forced-choice task and a two-down one-up rule to estimate the threshold at 79% correct point on the psychometric curve (Levitt, 1971). The reference tone, which was presented once per trial, had a frequency of 500 Hz. The adaptive procedure was stopped after 15 reversals and the geometric mean of the last eight trials was recorded. Thresholds were derived from the average of five task repetitions.

### Stimulus presentation

The stimulus for the MEG/EEG recordings was a 120-ms synthesized speech syllable (/da/) with a fundamental frequency in the sustained vowel portion of 98 Hz. The stimulus was presented binaurally at 80 dB SPL, ~14,000 times in alternating polarity, through Etymotic ER-3A insert earphones with foam tips (Etymotic Research). For five subjects, ~11,000 epochs were collected due to time constraints. Stimulus onset synchrony (SOA) was randomly selected between 195 and 205 ms from a normal distribution. A separate run was collected of ~600 stimulus repetitions spaced ~500 ms apart, to record later waves of the slower cortical responses. To control for attention and reduce fidgeting, a silent wildlife documentary (Yellowstone: Battle for Life, BBC, 2009) was projected onto a screen at a comfortable distance from the subject's face. This film was selected for being continuously visually appealing; subtitles were not provided in order to minimize saccades.

### Neurophysiological recording and preprocessing

Two hundred and seventy-four channels of MEG (axial gradiometers), one channel of EEG data (Cz, 10–20 International System, averaged mastoid references), EOG and ECG, and one audio channel were simultaneously acquired using a CTF MEG System and its in-built EEG system (Omega 275, CTF Systems Inc.). All data were sampled at 12 kHz. Data preprocessing was performed with Brainstorm Tadel et al. (2011) and using custom Matlab scripts (The Mathworks Inc., MA, USA) as described in Coffey et al. (2016b), and in brief, below.

### FFR correlates of SIN accuracy

FFR-f0 strength was extracted from regions of interest (ROIs) in the auditory system (AC: auditory cortex, MGB: medial geniculate body of the thalamus, IC: inferior colliculus and CN: cochlear nucleus) using the MEG distributed source modeling approach described previously (for the specifications of each ROI, please see Methods in Coffey et al., 2016b). Using this approach, the amplitude of a large set of dipoles are used to map activity originating in multiple generator sites; these are constrained by spatial priors derived from each subject's T1-weighted anatomical MRI scan (Baillet et al., 2001; Gross et al., 2013), from which cortical sources and subcortical structures were prepared using FreeSurfer (Fischl, 2012). As reported in Coffey et al., anatomical data were imported into Brainstorm (Tadel et al., 2011), and the brainstem and thalamic structures were combined with the cortex surface to form the image support of MEG distributed sources: the mixed surface/volume model included a triangulation of the cortical surface (~15,000 vertices), and brainstem and thalamus as a three-dimensional dipole grid (~18,000 points). An overlapping-sphere head model was computed for each run; this forward model explains how an electric current flowing in the brain would be recorded at the level of the sensors, with fair accuracy (Tadel et al., 2011). A noise covariance matrix was computed from 1-min empty-room recordings taken before each session. The inverse imaging model estimates the distribution of brain currents that account for data recorded at the sensors. We computed the MNE source distribution with unconstrained source orientations for each run using Brainstorm default parameters. The MNE source model is simple, robust to noise and model approximations, and very frequently used in literature (Hämäläinen, 2009). Source models for each run were averaged within subject. We extracted a timeseries of mean amplitude for each ROI and for each of the three orientations in the unconstrained orientation source model, from which three spectra were obtained by first windowing the signal (5 ms raised cosine ramp), zero padding to 1 s to enable a 1 Hz frequency resolution, with subsequent fast Fourier transform, and rescaling by the proportion of signal length to zero padding. The spectra of the three orientations were then summed in the frequency domain to obtain the amplitude of each subject's neurological response at the fundamental frequency, which was detected by an automatic script; this is referred hereafter as the FFR-f0 strength.

We first evaluated correlations between SIN accuracy scores and FFR-f0 strength averaged across bilateral pairs of structures, using Spearman's rho (rs; one-tailed). Non-parametric statistics were used throughout as FFR-f0 measures were generally not normally distributed (using Shapiro-Wilk's parametric hypothesis test of composite normality, the null hypothesis was rejected for AC, CN, and IC bilateral averages), and one-tailed tests were used as our goal was to test the specific hypothesis that higher amplitude FFR-would be related only to better behavioral performance, as stronger or less degraded FFRs in the presence of noise have been reported consistently in the EEG literature (Cunningham et al., 2001; Parbery-Clark et al., 2009a,b, 2012a; Song et al., 2011), see also (Du et al., 2011). To correct for multiple comparisons, the false discovery rate (FDR) was controlled at δ = 0.05 where tests on multiple ROIs are used (Benjamini and Hochberg, 1995). The EEG equivalent of the FFR-f0 was also computed for comparison of sensitivity to behavioral measures. Correlations were computed between SIN accuracy and the left and right auditory cortex ROIs separately, as a lateralization effect in FFR-f0 strength and its relationship to measures of musicianship and fine pitch discrimination had been observed previously (see Figure 5c–e in Coffey et al., 2016b). We tested for a stronger correlation on the right than left side using Fisher's r-to-Z transformation (one-tailed, alpha = 0.05).

### Later cortical evoked responses

Event-related potentials (ERPs) within the 2–40 Hz band-pass filtered single-channel EEG data were obtained in order to establish a connection between previous FFR-ERP research that showed SIN sensitivity at ERP components P2 and N2 (Cunningham et al., 2001; Parbery-Clark et al., 2011a) and the MEG data. In order to maximize the interpretability of the results with respect to a large body of work that has used EEG-based measures of P2 amplitude (which may not be entirely equivalent to their magnetic counterparts due to differences in each technique's sensitivity to source orientation), the primary measure of ERP amplitude is based on EEG rather than MEG; MEG sources were localized for the same time period.

Although, SIN perception has also been found to be correlated with N1 latency and amplitude measures (e.g., Parbery-Clark et al., 2011a; Billings et al., 2013; Bidelman and Howell, 2016), N1 is strongly affected by the characteristics of the stimulus and its stimulation paradigm (Billings et al., 2011). In this paradigm, and using a single-EEG channel positioned at the vertex (Cz), we did not observe a clear N1 nor N2 from all subjects. We therefore took the amplitude of only P2 as a measure; this simpler metric occurs at a single time point and also allowed for a more straightforward comparison to and interpretation of the MEG equivalent. A researcher who was blinded to the subjects' FFR-f0 amplitudes and behavioral results at the time of measurement selected P2 wave peaks individually on ERP waves averaged across epochs for each subject (cortically processed; 2–40 Hz with −50 to 0 ms DC baseline correction; P2 was considered to be the strongest positive deflection within a ~40 ms window centered on the group grand average P2 at 183 ms). Amplitudes of these custom peaks were then correlated with SIN accuracy and FFR-f0 strength.

MEG evoked response fields (ERFs) on simultaneously recorded data were obtained in order to extend this work using distributed source modeling. The EEG cortical evoked response complex (ERP) elicited by the speech syllable /da/ consists of two positive waves at about 50–90 ms (“P1”) and between 170 and 200 ms (“P2” or “P1 prime”) and two negative waves at about 110 ms (“N1”) and after 200 ms (“N2” or “N1 prime”) (reviewed in Key et al., 2005); see also Cunningham et al., Figure 6 (Cunningham et al., 2001) and Musacchia et al., Figure 2 (Musacchia et al., 2008). For the purposes of this study we identified wave peaks in the ERP and ERF average at the group level for the SIN-sensitive P2 peak (183 ms), and at the earlier P1 component that has a well-known physiological origin in order to confirm the quality of data and validity of the analysis (60 ms; see **Figures 3A,C**).

### Origins of later cortical ERP components

To confirm that the MEG data could be used to localize areas that showed above-baseline activity at the group level, and to observe the origins of the SIN-sensitive P2 wave in relation to preceding and following ERP waves, we first computed cortical volume MNE models based on each subject's T1-weighted MRI scan in which the orientation of sources was uncontrained, but their location was constrained within the volume encompassed by the cortical surface. These models were normalized to the baseline period (−50 to 0 ms). We exported 10 ms time windows around each peak of interest (mean-rectified signal amplitude) and for the baseline (−50 to 0 ms) for statistical analysis in the neuroimaging software package FSL (Smith et al., 2004; Jenkinson et al., 2012). These source volume maps were co-registered to the subject's high-resolution T1 anatomical MRI scan (FLIRT, 6 parameter linear transformation), and then to the 2 mm MNI152 template (12 parameter linear transformation, Evans et al., 2012). Normalized difference images were created by subtracting the baseline images from those of the peaks of interest and calculating z-scores within each image (P1 > Baseline, P2 > Baseline). Permutation testing was used to reveal locations where the magnetic signal was greater during peaks of interest as compared with baseline [non-parametric one-sample *t*-test (Winkler et al., 2014); 10,000 permutations]. The family-wise error rate was controlled using threshold-free cluster enhancement as implemented in FSL (*p* < 0.01), after applying a cortical mask of the MNI 152 template with the brainstem and cerebellum removed (these latter structures were not included in the MEG source model).

### Comodulation of low and high frequency activity

We considered the spatial relationship between FFR-f0 generators and the source of the SIN-sensitive P2 wave by inspecting the FFR-f0 > Baseline and P2 > Baseline maps in the MEG data, and calculated Spearman's correlations between the FFR-f0 strength from each auditory cortex ROI (MEG) and the amplitude of the P2 wave measured with EEG.

### Musicianship enhancements

Twelve subjects reported varying levels of musical experience on a range of musical instruments [primary instruments: piano (6), guitar (2), flute (1), saxophone (1), trumpet (1), voice (1)], as obtained by self-report using the Montreal Music History Questionnaire (Coffey et al., 2011). Start ages ranged from 5 to 12 years, and total cumulative practice hours ranged from 1,000 to 16,000 h. We assessed correlations between SIN accuracy and total music practice hours and age of training start. We then evaluated the relationship between P2 amplitude in the EEG recording and musicianship, and between fine pitch discrimination skills and musicianship.

## Results

### Behavioral scores

The mean averaged SIN score was 56.3% (*SD* = 12.0). Subjects with finer pitch discrimination ability had statistically better SIN accuracy (one-tailed rs = −0.47, *p* = 0.018).

### MEG FFR-f0 strength is related to SIN throughout the auditory system

**Figure 1:**
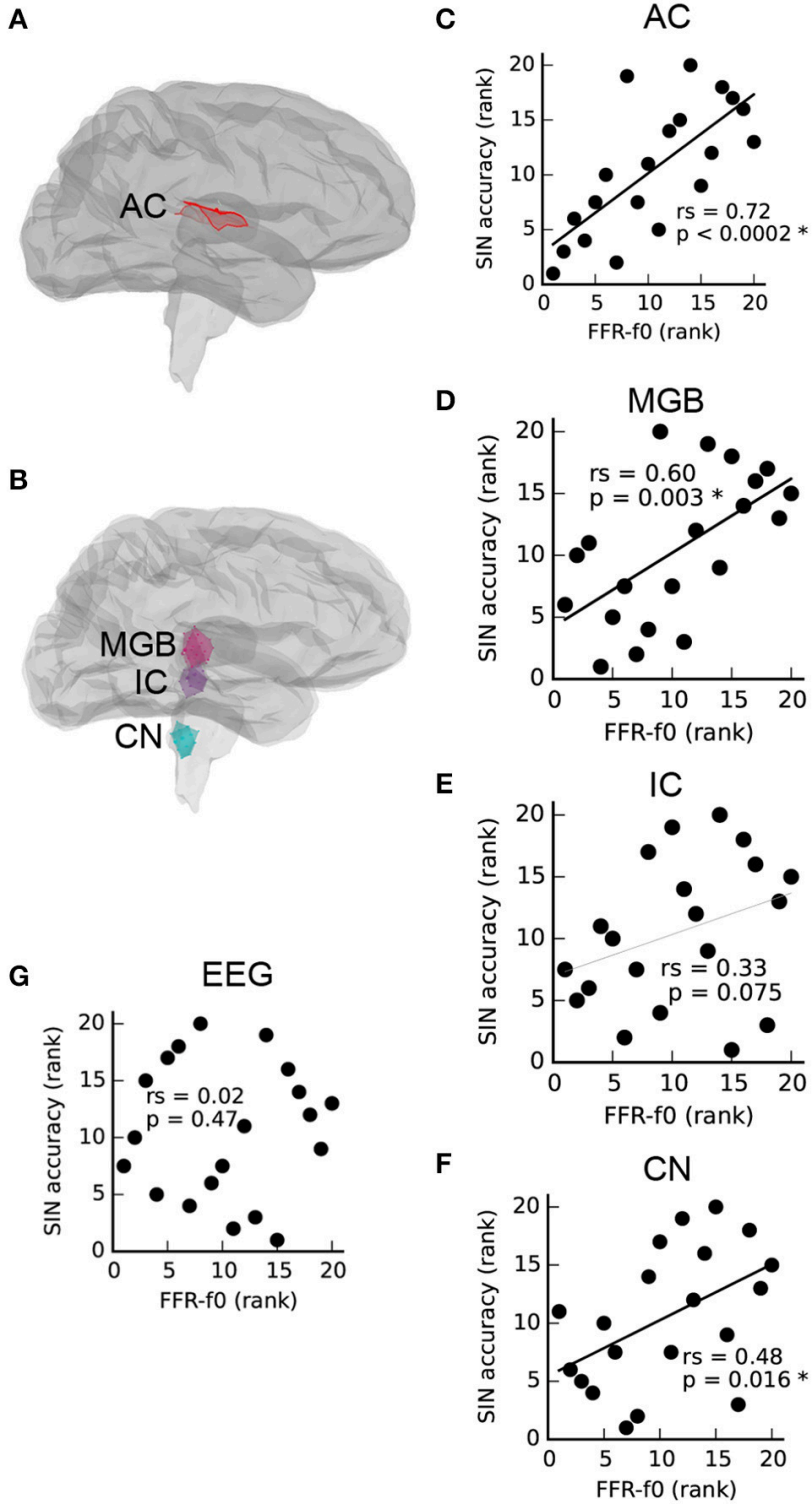
Correlations between FFR-f0 strength and speech-in-noise accuracy (SIN) within regions of interest (ROIs) in the auditory cortex **(A,C)**, and subcortical areas **(B,D–F)** as measured with magnetoencephalography (MEG) suggest that better SIN performance is related to better periodicity encoding throughout the auditory system. The FFR measured using electroencephalography at the vertex (Cz) is shown for comparison in **(G)**. AC, auditory cortex; MGB, medial geniculate body; IC, inferior colliculus; CN, cochlear nucleus. Correlations are calculated using Spearman's rho (rs).

As described by Coffey et al. (2016b), MEG is able to separate FFR activity arising from auditory cortex (Figure 1A), as well as brainstem and thalamus (Figure 1B). Relationships between FFR values measured via MEG from each ROI (averaged across left and right pairs) and SIN accuracy scores are presented in Figures 1C–F. A positive correlation between SIN accuracy and FFR-f0 strength was found at each of the four structures tested, statistically significant (FDR-corrected for multiple comparisons) in all but the inferior colliculus, where a similar trend was nonetheless noted. We did not find evidence of a relationship between the EEG-derived FFR-f0 and SIN accuracy (Figure 1G), nor did a relationship appear with the inclusion of age as a covariate (rs = 0.08, *p* = 0.37).

To confirm that the precautionary exclusion of the easiest SIN trials in which a ceiling effect was found was inconsequential with respect to the observed relationships between SIN and FFR-f0 strength, we recalculated the correlation between rAC FFR-f0 and SIN accuracy including all items (rs = 0.71, *p* = 0.0003; compare with the reported value with the exclusion, which is rs = 0.72, *p* < 0.0002). The general pattern of a positive correlation between rAC FFR-f0 and SIN accuracy was even replicated within the small subsets of easy (rs = 0.65, *p* = 0.001), medium (rs = 0.80, *p* < 0.0001), and hard items (rs = 0.57, *p* = 0.004), suggesting a robust relationship that is not highly sensitive to how the overall SIN accuracy score is calculated.

### The relationship between SIN and cortical FFR-f0 is lateralized

**Figure 2:**
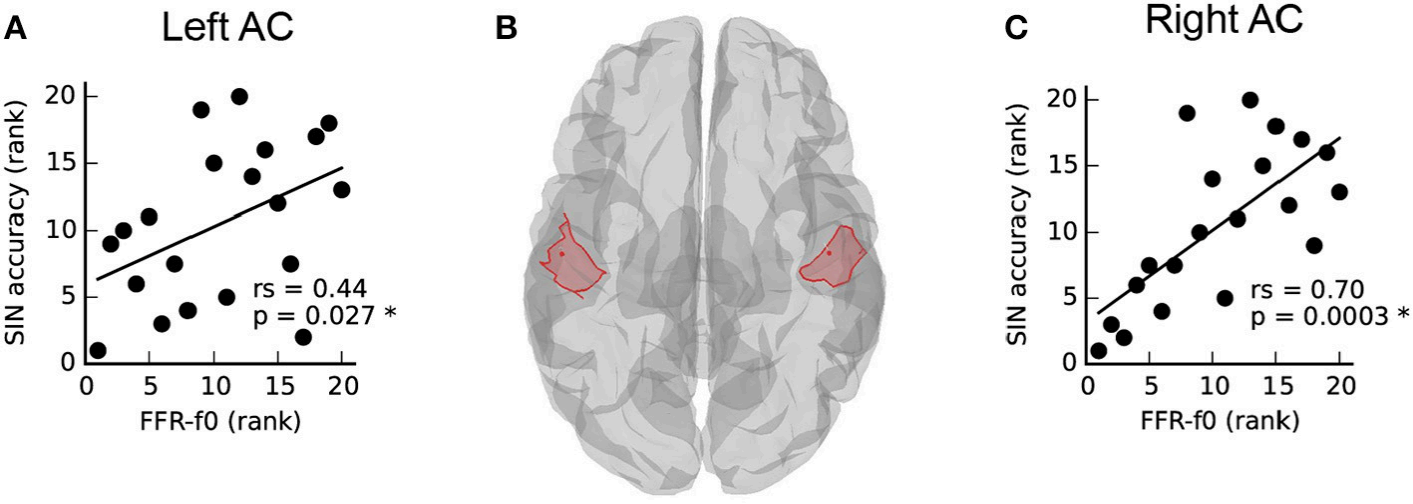
Asymmetry in the relationship between speech-in-noise (SIN) and cortical FFR-f0 representation **(A,C)** within left and right hemisphere auditory cortex ROIs, illustrated in **(B)**. Although a positive relationship between FFR-f0 strength and SIN is found in each hemisphere, it is significantly stronger in the right hemisphere (*Z* = −3.12, *p* = 0.001, one-tailed).

The relationship between SIN and FFR-f0 strength from auditory cortical ROIs in each hemisphere is depicted in Figure 2. SIN accuracy was related to the strength of the FFR-f0 in both hemispheres, but was numerically larger on the right. We directly compared the strength of these correlations using Fisher's r-to-Z-transformation (one-tailed), and found it to be stronger in the right hemisphere (*Z* = −3.12, *p* = 0.001; the correlation between the FFR-f0 strength across two hemispheres, which is used for statistical comparison of correlation strength, was rs = 0.89).

### Origins of later cortical ERP components

**Figure 3:**
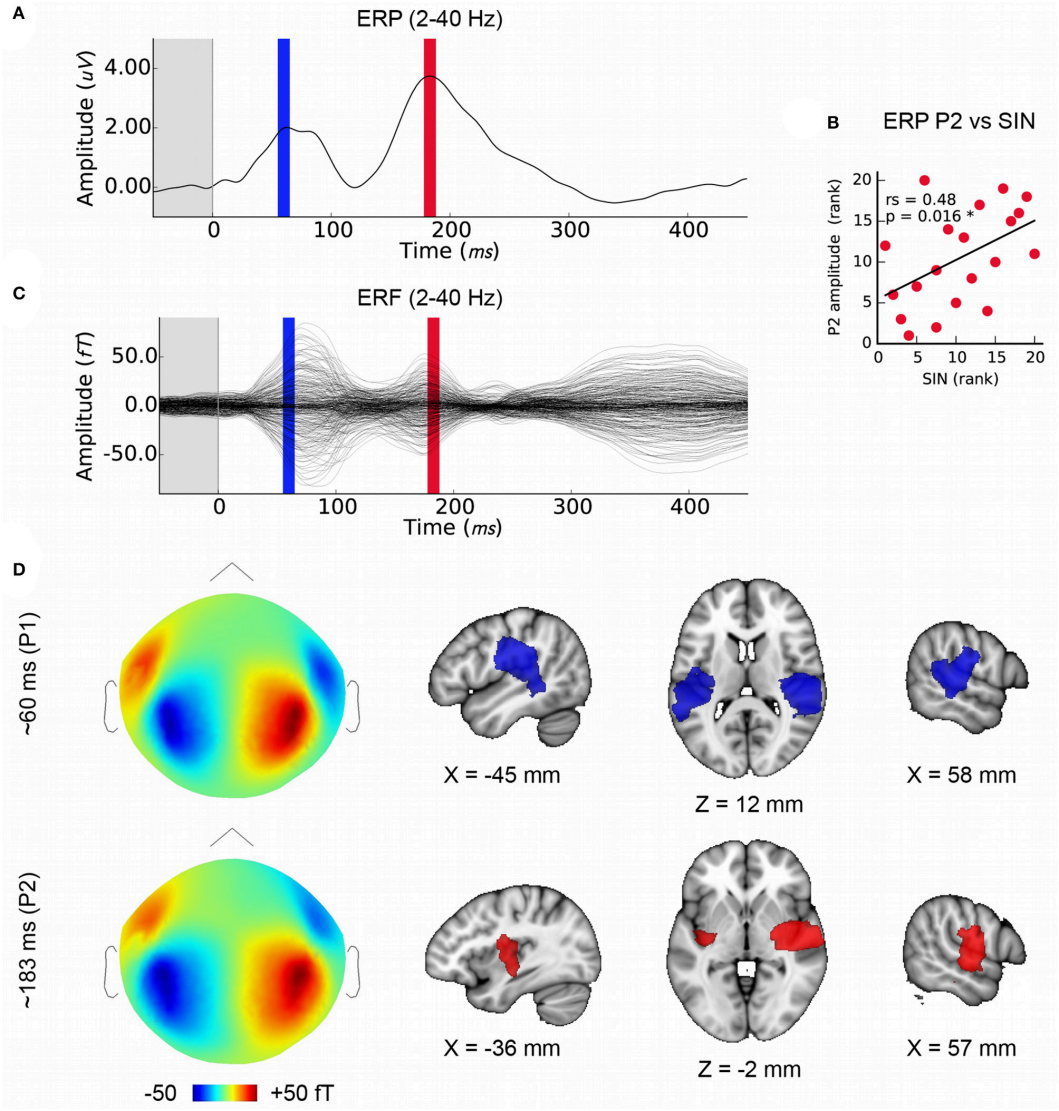
Later cortical evoked responses and their origins. **(A)** Time courses of the lower frequency evoked response potentials (ERPs) from EEG data with the time windows used for MEG source analysis marked (P1: blue, P2: red), and **(C)** evoked response fields (ERFs) from simultaneously recorded MEG data. Each is averaged over subjects (*N* = 20). **(B)** The amplitude of the P2 ERP wave peak (red) correlates with SIN accuracy. **(D)** Group-level MEG topographies (left, strength and polarity is indicated in the color bar below) and source analyses of P1 and P2 component origins using (1mm MNI space; cluster threshold; *p* < 0.005). Note that single-channel EEG data are used to derive P2 amplitudes in order to maximize interpretability with respect to previous work whereas source localization is performed on concurrent MEG data.

We confirmed that the MEG data analysis used here is suitable for localizing temporal lobe auditory activity at the group level using P1, which was known to originate in the primary auditory areas bilaterally (Figure 3D). Note that while we had selected the earliest maximum in the P1 wave in the EEG signal in order to capture primary auditory cortex activity (mean latency: 60 ms), the peak energy in the MEG signal is slightly later (~15 ms); nonetheless, visual inspection of the same analysis performed on a 10 ms window centered on 75 ms indicates that this analysis is not sensitive to minor variations in P1 window selection. The mean latency of P2, the second prominent positive EEG wave, was 183 ms (*SD* = 11 ms), and its mean amplitude was 4.1 uV (*SD* = 1.7). We confirmed, as previously reported by Cunningham et al. (2001) using a pediatric sample, that P2 amplitude was related to SIN accuracy in the current sample (Figure 3B) thus providing a basis for further investigating FFR-f0 and P2 relationships. P1 amplitude was not related to SIN accuracy (rs = 0.11, *p* = 0.33). We then identified the sources of magnetic activity that was concurrent with the EEG-derived P2 wave, which proved to be relatively more anterior, and right-lateralized (Figure 3D; colored areas depict significant clusters corrected for multiple comparisons; maps are thresholded to best expose the areas of strongest signal).

### Low and high frequency activity covary

**Figure 4:**
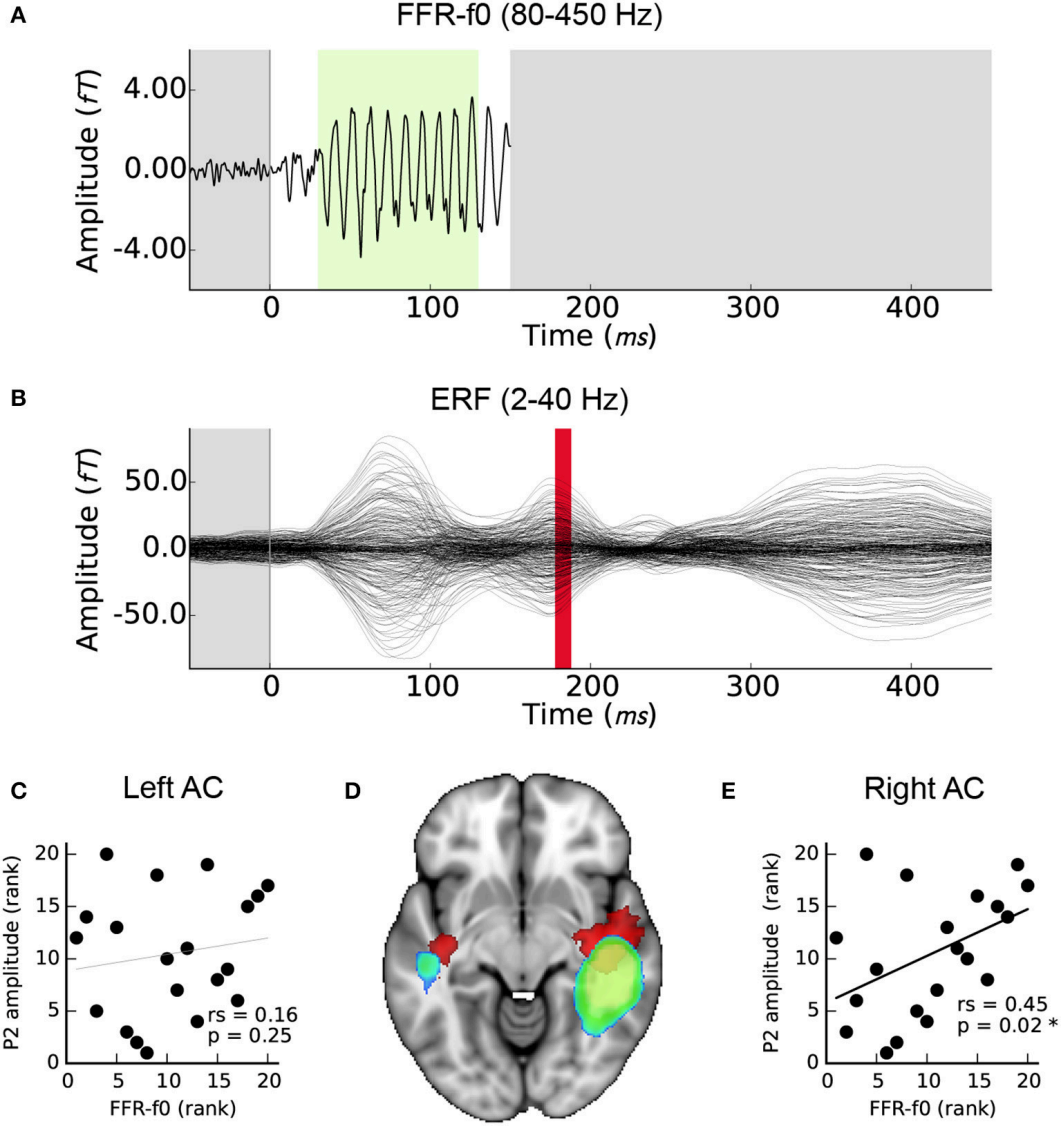
Relationship between FFR-f0 strength (blue-green) and P2 amplitude (red). **(A)** Time course of the FFR-f0 response, single channel (as presented in Coffey et al., 2016b; 80–450 Hz bandpass filtered; −50 to 150 ms window). The green portion indicates period over which FFR-f0 strength is calculated. **(B)** ERF over the same time period; these signals are separated by frequency band (2–40 Hz bandpass filtered) but P2 occurs after the FFR. Correlations between the FFR-f0 strength from the cortical ROIs as measured using MEG and the ERP strength at P2 as measured using EEG are shown for **(C)** the left and **(E)** the right auditory cortex. **(D)** Illustrates the relationship of the cortical origins of each signal (1mm MNI space; cluster threshold; *p* < 0.005).

Right but not left AC FFR-f0 strength was significantly related to P2 amplitude (Figures 4C,E). For completeness, we also calculated the correlation between the EEG FFR-f0 and P2 amplitude but it was not significant: rs = 0.23, *p* = 0.16). The magnetic equivalent of the P2 wave overlapped considerably with the FFR-f0 regions using a corrected significance-based threshold of *p* < 0.05. However, inspection of the centroid of each map showed that whereas the FFR-f0 sources were distributed in the posterior section of the superior temporal gyrus, the P2 wave's foci were more anterior (Figure 4D).

### Measures of musicianship

Twelve out of 20 subjects reported some level of musical training. We previously showed that FFR-f0 strength in the right AC is related to hours of musical training and age of training start in this sample (Coffey et al., 2016b). Here, we first confirmed that the correlation between AC FFR-f0 strength and SIN scores that we observed within the whole group was present within both the musician subgroup (averaged AC ROIs: rs = 0.67, *p* = 0.012) and the non-musical subgroup (averaged AC ROIs: rs = 0.86, *p* = 0.005); these results represents an internal replication in independent groups and suggest that the sample size used in this study is sufficiently large to be sensitive to the statistical relationships of interest. Musical enhancements in behavioral and neurophysiological measures reported in this study are summarized in Table 1. Among those with musical experience, earlier start ages significantly correlated with better SIN scores, but the correlation between total practice hours and SIN showed only a non-significant trend. P2 wave amplitude correlated with practice hours, but not the age of start. We also found a significant correlation between hours of musical training and fine pitch discrimination ability, as expected, and a non-significant trend between start age and fine pitch discrimination ability.

## Discussion

In this study, we aimed to clarify whether individual differences observed in fundamental frequency (f0) encoding in the auditory system of normal-hearing adults is related to SIN perception. Toward this end, we filtered the neural responses in two frequency bands in order to isolate the higher-frequency 98 Hz f0 within the FFR, and the lower-frequency cortical responses (2–40 Hz), and we compared their strength, spatial origins, and relationships to behavioral and musical experience measures.

We first showed that the strength of the MEG-based FFR-f0 attributed to structures throughout the ascending auditory neuraxis, including the auditory cortex in each hemisphere, is positively correlated with SIN accuracy (Figures 1C–F), suggesting that basic periodic encoding is enhanced throughout the auditory system in people with better ability to perceive speech under challenging noise conditions. Although, the IC vs. SIN relationship falls short of significance, the trend is in the predicted direction (rs = 33, *p* = 0.075); it is therefore most parsimonious to assume that we failed to observe the relationship strongly here for reasons related to noise in the data rather than that the strength of the pitch representation is uncorrelated at a midpoint location between nuclei through which the same information passes.

Importantly, the principal similarity between the conditions under which the neurophysiological measurement was made (i.e., passive listening in silence) and the behavioral measure [i.e., deciphering speech in sentences, similar to the clinical HINT task (Nilsson, 1994)] was the presentation of an auditory speech stimulus. Naturalistic SIN situations offer multiple cues, many of them redundant, that can result in flexibility in how the task is solved. This is true to a lesser degree of clinical tests of SIN (including the HINT variant used here), which approximate the naturalistic experience of perceiving speech masked by sound, but lack factors that are important in real-life conversations such as familiarity with the talker (Nygaard et al., 1994; Souza et al., 2013), visual cues (Zion Golumbic et al., 2013), and context- and listener-dependent adaptations of the speaker (Lombard, 1911). Tasks that have been used to study the neural correlates of SIN perception range in cue-richness from natural language comprehension in daily life, to intermediate tasks like the sentence-in-noise and word-in-noise measures, to discrimination of phonemes from among a restricted set of possibilities (Du et al., 2014), and finally to passively listening to single sounds with or without masking noise; different types of noise such as energetic maskers and informational maskers have also been investigated (Swaminathan et al., 2015). Because the effects of variations in paradigm design are unclear (Wilson et al., 2007), [for example the HINT may be presented with or without spatial cues (Nilsson, 1994)], this diversity may be contributing to confusion in SIN literature, for example regarding the possibility of a using musical training to improve SIN skills (Coffey et al., 2017). A systematic analysis of SIN task requirements and differences in their neural correlates may be helpful to clarify these issues [e.g., by using task decomposition (Coffey and Herholz, 2013)].

According to such an approach, our neurophysiological recording paradigm did not explicitly engage auditory working memory and it did not require attention, which instead was directed to a silent film. Although, top-down processes might conceivably act spontaneously even in the presence of such a distraction, the speech sounds were presented in perfect clarity and did not offer more than one stream of information upon which top-down mechanisms of auditory stream analysis such as stream segregation (Başkent and Gaudrain, 2016) or selective auditory attention (Song et al., 2011; Lehmann and Schönwiesner, 2014) might act.

Because we have eliminated cues that might be used by these higher-level processes that are known to affect SIN, either by enhancing the incoming signal (e.g., Lehmann and Schönwiesner, 2014) or at later linguistic/cognitive processing stages, any residual relationship between basic sound encoding (measured in silence) and scores on a high-level SIN task is most parsimoniously explained by the benefits of better lower-level sound encoding. Thus, we believe that the correlations we observed at brainstem, thalamic and cortical levels are best interpreted as reflecting these low-level enhancements.

Amplitude differences in the P2 cortical component and in FFR-f0 strength have been related to SIN perception differences between normal and learning-disordered children when recorded in noise (Cunningham et al., 2001). P2 amplitude also appears to be stronger in musicians (Shahin et al., 2003; Kuriki et al., 2006; Bidelman and Weiss, 2014; though this has not been observed in all studies Musacchia et al., 2008). P2, along with the P1 and the intervening negativity, is affected by stimulus parameters including frequency, location, duration, intensity, and presence of noise (reviewed in Alain et al., 2013; see also Ross and Fujioka, 2016), is affected by short-term training (Lappe et al., 2011; Tremblay et al., 2014), and is correlated with language-related performance measures such as categorical speech perception (Bidelman and Weiss, 2014). While P2 amplitude is affected by repetition and predictability (Näätänen and Picton, 1987; Tremblay et al., 2014), the relationships we observe here between the P2 amplitude and SIN performance are unlikely to be unique to the high number of repetitions used in this experiment, as the relationship between P2 and SIN has been previously observed with fewer trials (Cunningham et al., 2001). In any case, any fatigue effects on P2 amplitude would be more likely to decrease rather than be the cause of a correlation with the FFR-f0 amplitude, which is known to be comparatively resistant to cognitive manipulation (Varghese et al., 2015) except under very specific conditions (Lehmann and Schönwiesner, 2014; Coffey et al., 2016a). Our findings therefore suggest that the underlying processes are critical to sound representation generally, though the nature and roles of component processes represented in the P2 and their relationships to oscillatory brain networks are still being clarified (e.g., Ross et al., 2012; Ross and Fujioka, 2016).

We found that variability in P2 amplitude correlated with inter-individual differences in SIN ability (Figure 3B) and in FFR-f0 strength (Figures 4C,E), despite that the responses were measured only in quiet conditions and in a normal healthy adult population. These results suggest that previously reported relationships may be present as a continuum in the population and even in optimal listening conditions. The MEG-FFR technique may allow us to more consistently observe behavioral and experience-related relationships with FFR-f0 strength in less challenging listening conditions as compared with the EEG-FFR. The EEG-FFR is likely a composite from several subcortical and cortical sources (Herdman et al., 2002; Kuwada and Anderson, 2002; Coffey et al., 2016b; King et al., 2016; Zhang and Gong, 2016; Tichko and Skoe, 2017). In recent work, we compared two common single-channel EEG montages (Cz-mastoids and Fz-C7) and found that while FFR-f0 strength in each montage (measured simultaneously) was moderately correlated, a large proportion of variability was unaccounted for and the two methods differed in their sensitivity to a behavioral measure of interest (Coffey et al., 2016a). This observation suggests that differences in individuals' head and brain geometry may sometimes obscure EEG-FFR vs. behavioral relationships, possibly due to interference from source summation at a given point of measurement.

The spatial resolution of MEG source imaging may help to clarify the auditory processes that generate the ERP and ERF components, which have a long history in auditory neuroscience yet have predominantly been studied at the sensor level or using simpler models. We used distributed source modeling based on individual anatomy to localize the sources of each ERP/ERF wave (Figure 3D) with a view to confirming the localization of the wave of interest. The P1 wave originated bilaterally in the primary auditory areas, as expected (Liégeois-Chauvel et al., 1994; Key et al., 2005). P2 appeared to be right-lateralized and comparatively more anterior along the superior temporal plane as compared to the P1 signal. This observation is generally consistent with previous work (Alain et al., 2013), but contrasts with an analysis of equivalent current dipoles that suggested a more posterior and medial source for the P2 (Shahin et al., 2003). However, both the stimulation and analysis (e.g., use of the standard brain rather than individual anatomy) vary considerably between these studies, making this difference difficult to interpret.

The relatively more anterior location of the P2 compared to the FFR generators (Figure 4D) could be explained by a right-lateralized anterior flow of pitch-relevant information that supports SIN processing, although future work will be needed to clarify whether the relationship between periodic encoding and later waves is causal in nature, or if these different frequency bands represent neural activity in parallel processing streams in neighboring neural populations. Furthermore, as mentioned previously, earlier ERP components such as N1 have previously been related to SIN perception (Billings et al., 2013; Bidelman and Howell, 2016). Although, we were not able to measure it with the experimental design used here, it is possible that a relationship exists between FFR and slower cortical activity earlier than P2. A combination of multichannel EEG and MEG, and a stimulation paradigm that is optimized to clearly and consistently evoke each ERP waveform may be highly informative as to how basic auditory information is separated and streamed to other cortical areas in order to accomplish different auditory tasks, using the spatial information in MEG data or a combinations of EEG and fMRI data.

The strength of signal generators in the right hemisphere was stronger for both the FFR-f0 and P2 waves (Figures 4A,B,D). We found a positive correlation between P2 amplitude (measured with EEG at Cz) and MEG FFR-f0 in the right hemisphere (but not left; Figures 4C,E). These results corroborate previous work suggesting that the right auditory cortex is relatively specialized for pitch and tonal processing (Zatorre et al., 1994; Patel and Balaban, 2001; Patterson et al., 2002; Schneider et al., 2002; Hyde et al., 2008; Mathys et al., 2010; Albouy et al., 2013; Andoh et al., 2015; Herholz et al., 2015; Cha et al., 2016), as well as reports of experience-sensitive relationships between FFR-f0 and lower frequency auditory evoked responses (Musacchia et al., 2008; Bidelman et al., 2014). It has been proposed that subtle differences in neural responses early in the cortical processing stream may lead to distinct functional roles for higher level processes out of a need for optimization, in particular a right-hemisphere bias for periodicity and left-hemisphere bias for fine temporal resolution (Zatorre et al., 2002). It is likely that other aspects of SIN processing, particularly those related to linguistic cues, are also relatively more left-lateralized, and that overall laterality of SIN-related processing may depend on the degree to which a given paradigm engages combinations of neural systems (Shtyrov et al., 1998, 1999; Laine et al., 1999; Du et al., 2014, 2016; Bidelman and Howell, 2016). The relatively stronger temporal representation of periodicity in the right hemisphere may be the underlying reason for which we had previously observed a hemispheric asymmetry in the FFR-f0 and its relationship to fine pitch discrimination ability, hours of musical practice, and age of training onset (see Figure 5c–e in (Coffey et al., 2016b); statistics are reported in the present work in Table 1). The present results are congruent with these hypotheses.

Periodicity encoding is related to pitch information (Gockel et al., 2011), which is one of several cues that the brain can use to separate streams of auditory information (Bregman, 1994; Moore and Gockel, 2002) and which is useful to help distinguishing one vowel sound from another (Chalikia and Bregman, 1989; Summerfield and Assmann, 1999) and can help speech segregation at the sentence level (Brokx and Nooteboom, 1982). We previously showed that fine pitch discrimination skills are correlated with FFR-f0 strength in the right auditory cortex (Coffey et al., 2016b). Here, we add that discrimination thresholds correlate with SIN accuracy, and that SIN accuracy is related to periodic encoding in the auditory cortex. Together, these results support a mechanistic explanation for SIN enhancement via better pitch processing leading to better stream segregation (Anderson and Kraus, 2010a). This explanation can also account for musician advantages in SIN, as well as the inconsistency with which it is observed across studies, whose design may emphasize other, non-periodic SIN cues. In the subset of subjects who reported having had musical training, measures of the extent and timing (age of start) of musical practice were related to behavioral measures of SIN accuracy and fine pitch discrimination, and were paralleled in physiology by relationships to FFR-f0 and P2 amplitude. Experience-dependent plasticity therefore likely tunes FFR-f0 strength and tracking ability as suggested by several prior studies (Musacchia et al., 2007; Song et al., 2008; Bidelman et al., 2011a; Carcagno and Plack, 2011). Stronger periodicity encoding might thereby account in part for a musician advantage. However, other top-down factors are also at play in SIN perception, including auditory working memory, long-term memory, and selective attention. Each of these may be influenced by experience, and other peripheral and central factors (Anderson et al., 2013b). These latter factors would likely be related to top-down effects originating in extra-auditory cortical areas such as motor and frontal cortices (Du et al., 2014) whereas the feed-forward mechanisms we emphasize here likely represent neural modulations within ascending neural pathways including brainstem nuclei, thalamus, and cortex. Any or all of these mechanisms may be enhanced by musical training.

We propose that whether a musician advantage in SIN perception is observed or not in a given study may depend on interactions between the current state of the auditory system and the specific cognitive demands of the SIN task used in the study. Specifically, performance can depend on (1) the cues offered to the listener in the SIN paradigm [e.g., spatial cues and degree of information masking; (Swaminathan et al., 2015)]; (2) the degree to which an individual's experience has enhanced representations and mechanisms related to the available cues and caused them to be more strongly weighted; and (3) how well individuals can adapt to use alternative cues and mechanisms when one or more cues becomes less useful either through task differences like levels of noise (e.g., Du et al., 2014) or due to physiological deterioration (Anderson et al., 2013b).

## Conclusion

In this study we present novel evidence that the quality of basic feed-forward periodicity encoding is related to the clinically relevant problem of separating speech from noise signals, and musical training. Specifically, in the absence of contextual cues and task demands and given a measurement tool that is sensitive to signal sources (i.e., MEG), enhancements in periodic sound encoding throughout the auditory neuraxis were correlated with better SIN ability in an offline task of sentence perception. This effect was observed to be stronger in the FFR signal localized to the right auditory cortex, and was related to slower cortical P2 wave amplitude measured by EEG, which is concurrent with activity in the right secondary auditory cortex measured with MEG and suggests an anterior flow of pitch-related information. Musicians show an advantage related to FFR strength, suggesting a possible role of experience. Our results suggest that inter-individual differences in neural correlates of basic periodic sound representation observed within the normal-hearing population (Ruggles et al., 2011; Coffey et al., 2016a) may in part be responsible for the surprising variability in SIN perception observed inter-individually. This work sketches in the anatomical and temporal properties of a stream of pitch-relevant information from subcortical areas up to and beyond right primary auditory cortex. More work is needed to explore exactly how this information is routed and subsequently used by higher-level networks. We conclude that better sound encoding likely improves SIN perception through better representation of periodicity, which in turn leads to better stream segregation. Importantly, these findings may have practical implications for people suffering from difficulty with SIN perception because basic sound encoding fidelity has been shown to be malleable via training; these results therefore support efforts to develop training-based treatment strategies (Bidelman and Alain, 2015) to improve people's participation in social, vocational, and educational activities (Anderson and Kraus, 2010b).

## Ethics statement

This study was carried out in accordance with the recommendations of the Montreal Neurological Institute Research Ethics Board with written informed consent from all subjects. All subjects gave written informed consent in accordance with the Declaration of Helsinki. The protocol was approved by the Montreal Neurological Institute Research Ethics Board.

## Author contributions

EC, SH, SB, and RZ designed the experiment, AC and EC collected the data, EC analyzed the data, and EC, SH, SB, and RZ wrote the paper.

### Conflict of interest statement

The authors declare that the research was conducted in the absence of any commercial or financial relationships that could be construed as a potential conflict of interest.
